# Are CT-Based Finite Element Model Predictions of Femoral Bone Strengthening Clinically Useful?

**DOI:** 10.1007/s11914-018-0438-8

**Published:** 2018-04-14

**Authors:** Marco Viceconti, Muhammad Qasim, Pinaki Bhattacharya, Xinshan Li

**Affiliations:** 1 Department of Mechanical Engineering, University of Sheffield, Sheffield, UK; 2 INSIGNEO Institute for in silico Medicine, University of Sheffield, Sheffield, UK; 3 Insigneo Institute for in silico medicine, University of Sheffield, Pam Liversidge Building, Sheffield, S13JD UK

**Keywords:** Hip fracture, Computed tomography, Subject-specific finite element models, Cost-benefit

## Abstract

**Purpose of Review:**

This study reviews the available literature to compare the accuracy of areal bone mineral density derived from dual X-ray absorptiometry (DXA-aBMD) and of subject-specific finite element models derived from quantitative computed tomography (QCT-SSFE) in predicting bone strength measured experimentally on cadaver bones, as well as their clinical accuracy both in terms of discrimination and prediction. Based on this information, some basic cost-effectiveness calculations are performed to explore the use of QCT-SSFE instead of DXA-aBMD in (a) clinical studies with femoral strength as endpoint, (b) predictor of the risk of hip fracture in low bone mass patients.

**Recent Findings:**

Recent improvements involving the use of smooth-boundary meshes, better anatomical referencing for proximal-only scans, multiple side-fall directions, and refined boundary conditions increase the predictive accuracy of QCT-SSFE.

**Summary:**

If these improvements are adopted, QCT-SSFE is always preferable over DXA-aBMD in clinical studies with femoral strength as the endpoint, while it is not yet cost-effective as a hip fracture risk predictor, although pathways that combine both QCT-SSFE and DXA-aBMD are promising.

## Introduction

According to the standard of care accepted in most countries, the risk for a given patient to experience a fragility hip fracture is determined indirectly by measuring the areal bone mineral density (aBMD) at the hip region using dual X-ray absorptiometry (DXA). This information is then combined with clinical risk factors (such as age, gender, weight, height, previous fractures, smoking, etc.) in epidemiological models such as FRAX that provide an estimate of the absolute risk of fracture over 5 or 10 years [1]. Approximately half of the patients who face a hip fracture are considered at low risk with these risk predictors [2]. This alone would suggest that we need better ways to estimate the risk of hip fracture in fragile elderlies.

The strength of a bone (the intensity of the force loading the bone in a certain direction that is required to fracture it) can be measured only invasively and destructively. However, since 1985 when it was first described in the literature [3], considerable effort has been spent in developing subject-specific finite element models derived from quantitative computed tomography (QCT-SSFE) to predict non-invasively bone strength. Today QCT-SSFE can predict such strength with excellent accuracy, possibly higher than that provided by hip DXA-aBMD [4]. If this is true, the strength estimated by QCT-SSFE models should provide a better predictor of the hip fracture risk assessment, compared to the current standard of care, DXA-aBMD.

Despite this, in a paper reporting the conclusions of the International Society for Clinical Densitometry (ISCD) 2015 Position Development Conference [5], Zysset et al. wrote: “Femoral strength as estimated by QCT-based FEA is comparable to hip DXA for prediction of hip fractures in postmenopausal women and older men”. In the rationale, while they acknowledged that “From biomechanical tests in vitro, FEA is overall a better surrogate of spine and hip failure load than DXA aBMD”, the results published up to 2014 [6–10] could not find significant differences between QCT-SSFE strength and DXA-aBMD in discriminating between fractured and non-fractured patients. More recent studies propose methodological improvements that may modify these conclusions [11••, 12••]. However, even in that case it is unclear if such differences are large enough to justify, for each possible clinical use case, a change of technology.

The aim of the present study is to review the most recent relevant literature to establish if, and under which conditions, it is convenient (in terms of effectiveness, cost, risk, and availability) to replace the current DXA-aBMD as a predictor of the risk of hip fracture with the femoral strength predicted by QCT-SSFE models.

Due to space limitations, in this review, we will not consider a third approach, where subject-specific finite element models are generated from DXA images (DXA-SSFE) [13, 14]. This method has an accuracy in predicting hip strength somehow intermediate between DXA-aBMD and QCT-SSFE [15].

### Accuracy of DXA-aBMD vs QCT-SSFE in Predicting Bone Strength

QCT-SSFE models can accurately predict the deformation induced in a cadaveric femur by any loading. In an extensive study where over 600 independent bone deformation measurements were made, QCT-SSFE models generated using a modelling technology (hereinafter referred to as *CT2S*) were found to predict the deformation of human femurs (induced by external loads) with a root mean squared error (normalised by the peak measured strain) of only 7% [16]. Considering the complexity of biomechanical deformation of mineralised tissues, it seems unlikely that much higher predictive accuracies can be expected in the future. Since aBMD cannot be used to predict strains, no comparison is possible for this indicator.

When QCT-SSFE models were used to predict the strength of human cadaver femurs, there is no consensus in the literature on how to express the predictive accuracy. Since the measured and the predicted values express the same quantity using the same unit of measurement, a good representation of average error of the predictor is the Standard Error of the Estimate of the linear regression between measured and predicted values, normalised by the average measured strength (%SEE). When the results are analysed with this error estimator, four validation studies published by four research groups using different but similar QCT-SSFE modelling technologies published in the last 6 years, involving 184 cadaveric femurs, reported a %SEE between 15 and 16% [4, 17–19]. Given all these models used strain as a predictor of fracture, and that fracture is a much more complex phenomenon to predict than strain, an error in predicting strength that is double of that for predicting strain seems reasonable and difficult to reduce significantly any further in the future.

As aBMD measurements are only indirect predictors of strength, the error metric most comparable to the %SEE used for QCT-SSFE models is probably the standard error of the Regression (%SER) between the measured strength and measured aBMD, again normalised by the average measured strength. Looking at five large studies [4, 20–23], the average error of aBMD as predictor of femoral strength, as measured by %SER over 300 femurs was on average 22% (range 19–23%).

These results suggest that QCT-SSFE can predict femoral strength from QCT images with an accuracy that is 6–7 pp.^1^ higher than that provided by aBMD. The good reproducibility of the error estimate for both QCT-SSFE and DXA-aBMD between research groups suggest the methods are reasonably mature.

One possible additional improvement is to account for the tissue anisotropy (which is not detectable at the resolution of clinical QCT) using a population-based statistical atlas obtained with high-resolution micro-CT imaging on cadaveric bones [24]. A recent study [25] suggested that this approach could improve the average accuracy of QCT-SSFE to predict bone strength by another 3 pp (compared to %SEE of 15–16% without anisotropy). This modification would bring the improvement of QCT-SSFE over aBMD to a significant 10 pp. However, the study did not calculate the accuracy against experimental results, but against the prediction of another FE model generated from higher resolution data. Thus, while this approach is promising, for the time being the clinical accuracy of QCT-SSFE methods in predicting bone strength should be considered to be 6–7 pp higher than that of aBMD.

### Evaluation of Clinical Accuracy

The issue of how to compare the clinical performance of these predictors for hip fracture risk assessment is complex. There are two different questions that need to be answered:

- For a given predictor, given a group of patients, some who at the time of enrolment already had a hip fracture, and some who at the same time did not, how accurately can the predictor separate the fractured patients and non-fractured ones? This will be referred to hereinafter as *discrimination accuracy*.
- Given a group of patients at risk but who initially had no hip fractures, how accurate can each predictor identify those patients who experienced a hip fracture within the following 5 or 10 years after the DXA and QCT examination were performed? This will be referred to hereinafter as *prediction accuracy*.

If the clinical use case involves the evaluation of a relative change in strength between two or more controls of the same subject at different time points, what matters is the discrimination accuracy. If the clinical use case is to predict the absolute risk of fracture, the question is whether the strength as predicted today can inform what happens tomorrow; this also involves the progression of the disease over time.

Following this logic, discrimination accuracy could be evaluated with data collected with cross-sectional studies [26, 27], while prediction accuracy would require data from longitudinal studies where patients are scanned at baseline and then followed-up for years [6, 7, 9, 10]. However, because of the modest incidence of hip fractures in the general population, longitudinal studies are very difficult and expensive to conduct and rarely produce well-paired fractured/control cohorts over age, height, and weight. Also, the odds ratios for the DXA-aBMD predictor reported in previous cross-sectional studies are in the same range of those obtained from longitudinal studies. This suggests that DXA-aBMD and QCT-SSFE strength are both predictors of the absolute risk of fracture at 5 (ARF5) or 10 years (ARF10). Thus, it seems plausible to evaluate DXA-aBMD and QCT-SSFE prediction accuracy also with data from cross-sectional studies.

Even cross-sectional studies have their own limitations. The most important is that a predictor cannot be calculated for the fractured hip; normally the predictor value for the controlateral intact hip is used instead. Of course, this is an additional potential source of bias; while on average such differences as mechanically negligible, in some random cases differences can be significant [28].

### Discrimination Accuracy of DXA-aBMD vs QCT-SSFE

The ISCD 2015 paper reached the conclusion that QCT-SSFE strength is not significantly better than DXA-aBMD in predicting hip fractures on the basis of five studies [6–10]. All these studies do have some methodological limitations, when compared to the current state of the art.

Firstly, all these studies used Cartesian meshes, where each voxel of the QCT 3D image is converted into a hexahedral finite element. While Cartesian mesh offers many advantages, it is unquestionably less accurate in predicting stresses and strains than modelling methods that use smooth meshes obtained from segmented CT images [29, 30]. How much this loss of accuracy impacts on the accuracy of strength prediction depends on a number of factors, with the most important ones being the constitutive equation and failure criterion [18, 31].

Secondly, all five studies used proximal femur QCT scans, limited to the metaphyseal and epiphyseal portions of the femur. A recent study has shown that this may introduce uncertainties in the anatomical orientation of the femur, thus making it difficult to reproducibly define the loading directions if not properly corrected using additional atlas information [12••].

Thirdly, three of the studies that supported ISCD conclusions [6, 7, 9] used only a single loading direction to predict the strength under side-fall conditions; one [8] used three, and the other [10] used eight. A study published in 2014, but not included in the ISCD review [11••], confirmed that a single-load side fall strength predicted by QCT-SSFE was not significantly more accurate than DXA-aBMD. But when the minimum strength under side-fall conditions (MSS) out of 10 simulated fall directions was used instead, QCT-SSFE yielded a significant improvement over DXA-aBMD [11••]. In this study, the improvement was quite dramatic: in a cohort of 22 fractures and 33 controls, they found that the total femoral DXA-aBMD Area under the ROC curve (AUC) was 0.79, that of single-load QCT-SSFE was 0.77, but that of multiple-load QCT-SSFE was 0.88. One limitation of this study was that fracture and control groups were not age-matched; when the same approach was used on a larger cohort pair-matched for age, height, and weight, the AUC of DXA-aBMD was found to be 0.75 and 0.79 with multiple-load QCT-SSFE [12••]. Lastly, the strength predicted by QCT-SSFE in side-fall has been recently reported to be highly sensitive to how precisely the impact force is modelled [32].

When all these improvements are included, the conclusions of the ISCD 2015 paper are indeed reversed. Recently, we evaluated the discrimination accuracy of the Insigneo CT2S modelling technology, that includes smooth-boundary meshes, anatomical orientation, 33 different side-fall directions, and refined non-linear boundary conditions on a retrospective cohort of postmenopausal women, formed by 50 cases of fragility hip fracture and 50 cases of controls (no fractures), pair-matched by age, weight, and height [27] (hereinafter referred as *Sheffield Cohort*) [33]. The AUC for the side-fall strength predicted by the QCT-SSFE model was 0.82, while that of aBMD was 0.75; thus, QCT-SSFE can separate fractured and non-fractured cases with an accuracy that is 7 pp higher than that of aBMD.

### Prediction Accuracy of DXA-aBMD vs QCT-SSFE

When these strength predictors are used not to classify, but to predict who is at risk, the definition of accuracy is immediate as soon as one has defined for each predictor a threshold value above/below which fracture is assumed to occur. Setting a threshold for DXA-aBMD is complex. However, if we use *T*-score based on DXA-aBMD, the WHO recommends an intervention if the *T*-score is equal of lower than − 2.5. For QCT-SSFE strength no such consensus is available. In a study on North American men, Orwoll et al. found all hip fractures occurred for a QCT-SSFE MSS strength of 2900 N or lower [6]. Based on this, Keaveny et al. proposed 3000 N as a threshold value [34]. This value was also used in a recent cost-effectiveness study on QCT-SSFE [35••]. Considering the gender differences, such threshold should probably be normalised for the average body weight that for North-Americans is 80.7 Kg [36]; this yields a threshold value of four-times the body weight (BW). When these thresholds were used on the Sheffield Cohort, we found a prediction accuracy of 66% for the *T*-score based on DXA-aBMD, and 73% for the MSS based on QCT-SSFE. So, again QCT-SSFE is 7 pp more accurate than DXA-aBMD. MSS prediction accuracy was reduced to 69% when the analysis was restricted to osteopenic patients.

### Comparison of Cost, Risk, and Complexity Between the Methods

Assuming these most recent implementations of QCT-SSFE technologies provide discrimination and prediction accuracies 7 pp better than DXA-aBMD, is this improvement sufficient to justify the higher risks involved with the higher costs, the higher radiation dose and the higher organisational complexity involved?

#### Cost

The cost of medical imaging varies considerably depending on the country, the healthcare provision model, etc. As we are interested to make a comparison in relative terms, here only official costs provided by the UK National Health Service (NHS) are used. Regarding the cost of the QCT-SSFE analysis service, we were able to recover this information only for two on-line services that provide QCT-SSFE modelling: the VirtuOst service provided by O. N. Diagnostics Inc.^2^ and the CT2S service provided by the Insigneo institute.^3^ The first is FDA-approved for clinical use, while the second can currently be used only for research. Currently for its clinical services, O. N. Diagnostics charges a cost for the BMD part of its test that is equivalent to the cost of a DXA exam, and provides the FEA analysis for free. The cost for FEA is not yet established but is expected to eventually be supported by clinical cost-effectiveness (Prof Tony Keaveny, personal communication). The CT2S service is tentatively priced to £250 per analysis, with a discount of 50% for non-sponsored studies run by not-for-profit organisations; here we will use the CT2S service figures as cost estimate. Assuming the use of non-reported imaging, the cost difference between DXA-aBMD and QCT-SSFE is £266 in UK (Table 1).

**Table 1:** Costs of DXA and CT from the official costing of the UK NHS; QCT-SFFE simulation service cost from the CT2S service

Exam	HRG code	2016–2017 tariff (unbundled, without reporting)
DEXA	RA15Z	£62
Computerised tomography scan, one area, no contrast, 19 years and over	RA08A	£78
QCT-SSFE analysis service	NA	£250

Adding £189 for the endocrinology visit (source: NHS official costs 2016/17), the total risk assessment costs are £251 for the DXA-aBMD pathway, and £522 for the QCT-SSFE pathway. It is assumed that a risk assessment visit is conducted every 2 years for all patients. In addition, it is also assumed the cost is £7200 for the pharmacological prevention (£60 per month per 10 years, Alendronic acid 70 mg tablets. Source: NHS Electronic Drug Tariff Jan 2018), £16,302 for the direct cost associated to a hip fracture [37], and £10,364 for the indirect costs [38]. In order to calculate the incremental cost-effectiveness ratio (ICER), we assumed an average quality-adjusted life year (QALY) of 0.91 for women over 55 with no fractures and 0.63 with a hip fracture [39].

#### Radiation Dose

The average effective radiation dose typically associated to a DXA exam is 0.001 mSv, whereas that of standard pelvis CT is 6 mSv. However, several measures can be adopted to reduce this effective dose. The whole femur CT scan protocol recommended by the CT2S service^4^ involves an effective radiation dose in the range of 1.9–4.8 mSv for males and 1.3–3.2 mSv for females. A recent study on the effect of reducing the X-ray energy on the predictive accuracy of QCT-SSFE of vertebral bodies suggests that even more aggressive reductions can be adopted without any significant loss of predictive accuracy [40].

#### Organisational Impact

For both CT2S and VirtuOst, the organisational impact involved with these services is minimal. The radiographer submits the DICOM files to the remote on-line service, and QCT-SSFE strength report is returned, typically via an email, in a time frame comparable to a standard radiology report (24–48 h). Assuming all this requires 10 min of an NHS grade 7 radiographer; this would cost approximately £8 per case.

### Appropriateness of QCT-SSFE in Clinical Research and Practice

#### Clinical Studies with Femoral Strength as Endpoint

It is common to conduct clinical studies where an intervention (physical or pharmacological) is evaluated against another intervention or the lack of thereof (i.e. placebo in the case of drug intervention), using bone strength as end point. Given its superior predictive accuracy, is it convenient to use QCT-SSFE in place of DXA-aBMD for strength estimation?

The first issue is the ethics involved with the use of higher radiation dose in a study for research purposes. This is a decision taken case by case by the local research ethics committee. However, considering a typical cohort of women over 50 years of age, these subjects have a general population risk of 2.8% of death related to a hip fracture [41]. If QCT-SSFE increases our ability to identify subjects at risk by 7 pp, 0.196% of the patients at risk of death for hip fracture would be treated instead. Considering current interventions avoid fractures in at least 40% of those who are treated [42], switching to QCT-SSFE would reduce the risk of death for complications associated to hip fracture by 0.0784%. Standard risk calculations^5^ suggest that a pelvic CT with an effective dose of 3.8 mSv would increase the risk of cancer by 0.0154%. Considering that the average mortality for cancer is 52%, the risk of death due the additional radiation could be estimated to increase by 0.0080%, which is much lower than the risk reduction QCT-SSFE seems to offer.

The second issue is the feasibility of the study, in relation to recruitment limits imposed by temporal, financial, operational or ethical constraints. Since QCT-SSFE predicts strength more accurately than aBMD, a simple statistical power calculation can be used to estimate the difference in cohort size that this improvement in accuracy would bring. The full detail of the calculations is provided in Table 2.

**Table 2:** Comparative use of QCT-SSFE and aBMD as a strength predictor in a clinical study. In order to detect a 20% difference in strength between two interventions, with significance level α = 0.05 and statistical power β = 80%, 245 patients need to be enrolled when using aBMD, while only 127 patients need to be enrolled when using QCT-SSFE

%SEE	aBMD	QCT-SSFE
	75%	82%
Average femoral strength (N)	3265	3265
Standard deviation of the predictor (N)	3054	2199
% difference in strength to be detected	20%	20%
α-error	0.05	0.05
□-power	80%	80%
Number of patients per group	123	64
Total number of patients in the study	246	128
Fixed costs for trial (£5000 patient)	£1,230,000.00	£640,000.00
Cost of imaging (£62 DXA; £78 CT)	£15,252.00	£9984.00
Cost of simulation (£250)	£-	£32,000.00
Total cost	£1,245,252.00	£681,984.00

In summary, only 127 patients are required to see differences of 20% in strength (statistically significant) between interventions with QCT-SSFE, in comparison to 245 patients using aBMD. While QCT-SSFE is more expensive than aBMD, the significant reduction in the cohort size will reduce the total cost of the trial. The key value is the fixed cost per patient, which in Table 2 is assumed to be £5000. According to a recent report in the Pharmaceutical Research and Manufacturers of America commissioned to Battelle, the average value is US$36,500 among trials of any phase or condition. Therefore, our assumption is relatively conservative.

^6^ https://tinyurl.com/Batelle-report

#### QCT-SSFE as Clinical Tool to Assess the Risk of Hip Fracture

With respect to this second use case, the risk–benefit analysis done for the first use case in relation to the increase in radiation dose, remains valid. Thus, the opportunity to adopt the strength predicted by QCT-SSFE models in place of the *T*-score measured with DXA needs to be seen entirely from a cost–benefit point of view. To this purpose, the costs were estimated based on a scenario of managing 1000 patients, who have been considered at risk of osteoporosis, referred to a secondary care specialist for 10 years, with three alternative clinical pathways (Table 3).

**Table 3:** Cost–benefit analysis of DXA-based *T*-score and QCT-SSFE pathways. We assumed sensitivity and specificity for both *T*-score and QCT-SSFE strength from the results for the Sheffield cohort reported above, and efficacy of treatment 40%

	DXA-*T*-score	QCT-SSFE	Dual pathway
Number of patients referred to secondary care	1000	1000	1000
Patients considered at risk and treated	367	602	633
Patients not treated	633	398	286
Patients who fracture under treatment	147	241	253
Patients who fracture without treatment	316	199	143
Total patients who fracture	463	440	396
Risk assessment costs	£1,255,000	£2,610,000	£1,899,184
Preventive pharma treatment cost	£2,644,898	£4,334,694	£4,555,102
Costs of hip fracture treatment (direct)	£7,552,151	£7,169,553	£6,454,261
Total cost hip fractures (direct costs)	£11,452,049	£14,114,247	£12,908,547
Costs of hip fracture treatment (indirect)	£4,801,282	£4,558,045	£4,103,298
Total cost hip fractures (total cost of care)	£16,253,331	£18,672,292	£17,011,845
Direct costs saved × 1000 patients	£-	*-£2,662,197.96*	*-£1,456,497.96*
Full costs saved × 1000 patients	£-	*-£2,418,961.22*	*-£758,514.29*
Fractures avoided by new pathway	–	23	67

Negative values in italic

In the first pathway, all patients who are osteoporotic according to the WHO definition (*T*-score ≤ − 2.5) are treated. In the second, all patients whose MSS ≤ 2551 N are treated. In the third (hereinafter called *dual pathway*), patients with *T*-score ≤ − 2.5 are treated; those with *T*-score > − 1.0 are not treated. Patients with − 2.5 < *T*-score ≤ − 1.0 are provided with further examination using the QCT-SSFE, and patients with MSS ≤ 2551 N are also treated. The percentage of patients that fall in each of these categories from the Sheffield Cohort was derived.

The use of QCT-SSFE as a risk predictor would always increase the costs and reduce the number of hip fractures. The QCT-SSFE pathway would involve a cost per QALY gained of £368,102, while the dual pathway would require a cost increase of £40,224 per QALY gained, which is considered not cost-effective according to the NICE thresholds. However, if we could reduce the cost of the simulation to £75 (~ US$100), the cost increase per QALY gained would be reduced to £14,656, which is considered cost-effective in most public healthcare systems.

## Discussion

This study revised the available literature to date comparing the accuracy of DXA-aBMD and QCT-SSFE in predicting bone strength measured experimentally on cadaver bones, as well as their clinical accuracy both in terms of discrimination and prediction. Based on this information, and the results obtained using a state-of-the-art QCT-SSFE technology called CT2S on a retrospective pair-matched cohort, some basic cost-effectiveness calculations were performed to explore the use of QCT-SSFE instead of DXA-aBMD in (a) clinical studies with femoral strength as endpoint, (b) predictor of the risk of hip fracture in low bone mass patients. We concluded that QCT-SSFE is always preferable over DXA-aBMD in clinical studies with femoral strength as the endpoint, while it is not yet cost-effective as a hip fracture risk predictor, although pathways that combine both QCT-SSFE and DXA-aBMD are promising.

Several recent studies conducted by different research groups using different QCT-SSFE technologies all indicate that QCT-SSFE is 6–7 pp more accurate than DXA-based aBMD (or *T*-score) in predicting femoral strength, in classifying fractured and non-fractured patients, and in predicting the risk of hip fracture.

In clinical trials using femoral strength as endpoint to evaluate the efficacy of an intervention in reducing the risk of hip fracture, this increase in accuracy can reduce as much as 50% of the cohort size that is required to recognise as statistically significant (*p* < 0.05) with 80% power, differences in strength between the two interventions being tested of 20% or greater. This involves additional cost for imaging and simulation, which are however offset by the reduction in the number of patients enrolled, and the associated fixed costs. In the light of these results, it is recommended to use QCT-SSFE as predictor of femoral strength in any clinical trial that uses strength as end point, instead of aBMD.

When the use of these predictors to support the decision to treat in secondary care settings was considered, a preliminary cost-benefit analysis suggested that a widespread adoption of QCT-SFFE would not be cost-effective. At the current cost for the CT2S service (£250), the use of QCT-SSFE on osteopenic cases only (dual pathway) is also not cost-effective. However, a recent cost-effectiveness study based on a state-transition microsimulation suggests that the combination of aBMD and QCT-SSFE is cost-effective, when the cost of simulation is assumed to be US$100 (~ £75) [35••]. Indeed, if we used that simulation cost, the dual pathway would become cost-effective.

## Footnotes

1 Hereinafter, we will use the % symbol to indicate the percent ratio between the value of interest and some average value that can be used to normalise it. Vice versa, we will use percentage point (annotated with the symbol pp) to indicate difference in percentage between two normalised values.

2
http://ondiagnostics.com

3
https://ct2s.sheffield.ac.uk

4
https://ct2s.sheffield.ac.uk/static/CT2SApp/multisim/CT2S_CTScan_Protocol.pdf

5
http://www.xrayrisk.com

## References

[1] Kanis JA, Harvey NC, Johansson H, Oden A, McCloskey EV, Leslie WD (2017). Overview of fracture prediction tools. J Clin Densitom.

[2] Wainwright SA, Marshall LM, Ensrud KE, Cauley JA, Black DM, Hillier TA, Hochberg MC, Vogt MT, Orwoll ES (2005). Study of osteoporotic fractures research G. Hip fracture in women without osteoporosis. J Clin Endocrinol Metab.

[3] Basu PK, Beall AG, Simmons DJ, Vannier M (1985). 3-D femoral stress analysis using CT scans and p-version FEM. Biomater Med Devices Artif Organs.

[4] Pottecher P, Engelke K, Duchemin L, Museyko O, Moser T, Mitton D, Vicaut E, Adams J, Skalli W, Laredo JD, Bousson V (2016). Prediction of hip failure load: in vitro study of 80 femurs using three imaging methods and finite element models-the European fracture study (EFFECT). Radiology.

[5] Zysset P, Qin L, Lang T, Khosla S, Leslie WD, Shepherd JA, Schousboe JT, Engelke K (2015). Clinical use of quantitative computed tomography-based finite element analysis of the hip and spine in the management of osteoporosis in adults: the 2015 ISCD official positions-part II. J Clin Densitom.

[6] Orwoll ES, Marshall LM, Nielson CM, Cummings SR, Lapidus J, Cauley JA, Ensrud K, Lane N, Hoffmann PR, Kopperdahl DL, Keaveny TM, for the Osteoporotic Fractures in Men (MrOS) Study Group (2009). Finite element analysis of the proximal femur and hip fracture risk in older men. J Bone Miner Res.

[7] Keyak JH, Sigurdsson S, Karlsdottir G, Oskarsdottir D, Sigmarsdottir A, Zhao S, Kornak J, Harris TB, Sigurdsson G, Jonsson BY, Siggeirsdottir K, Eiriksdottir G, Gudnason V, Lang TF (2011). Male-female differences in the association between incident hip fracture and proximal femoral strength: a finite element analysis study. Bone.

[8] Keyak JH, Sigurdsson S, Karlsdottir GS, Oskarsdottir D, Sigmarsdottir A, Kornak J, Harris TB, Sigurdsson G, Jonsson BY, Siggeirsdottir K, Eiriksdottir G, Gudnason V, Lang TF (2013). Effect of finite element model loading condition on fracture risk assessment in men and women: the AGES-Reykjavik study. Bone.

[9] Kopperdahl DL, Aspelund T, Hoffmann PF, Sigurdsson S, Siggeirsdottir K, Harris TB, Gudnason V, Keaveny TM (2014). Assessment of incident spine and hip fractures in women and men using finite element analysis of CT scans. J Bone Miner Res.

[10] Nishiyama KK, Ito M, Harada A, Boyd SK (2014). Classification of women with and without hip fracture based on quantitative computed tomography and finite element analysis. Osteoporos Int.

[11] Falcinelli C, Schileo E, Balistreri L, Baruffaldi F, Bordini B, Viceconti M, Albisinni U, Ceccarelli F, Milandri L, Toni A, Taddei F (2014). Multiple loading conditions analysis can improve the association between finite element bone strength estimates and proximal femur fractures: a preliminary study in elderly women. Bone.

[12] Qasim M, Farinella G, Zhang J, Li X, Yang L, Eastell R, Viceconti M (2016). Patient-specific finite element estimated femur strength as a predictor of the risk of hip fracture: the effect of methodological determinants. Osteoporos Int.

[13] Testi D, Viceconti M, Baruffaldi F, Cappello A (1999). Risk of fracture in elderly patients: a new predictive index based on bone mineral density and finite element analysis. Comput Methods Prog Biomed.

[14] Yang L, Parimi N, Orwoll ES, Black DM, Schousboe JT, Eastell R (2017). Association of incident hip fracture with the estimated femoral strength by finite element analysis of DXA scans in the Osteoporotic Fractures in Men (MrOS) study. Osteoporos Int.

[15] Johannesdottir F, Thrall E, Muller J, Keaveny TM, Kopperdahl DL, Bouxsein ML (2017). Comparison of non-invasive assessments of strength of the proximal femur. Bone.

[16] Schileo E, Dall'ara E, Taddei F, Malandrino A, Schotkamp T, Baleani M, Viceconti M (2008). An accurate estimation of bone density improves the accuracy of subject-specific finite element models. J Biomech.

[17] Dragomir-Daescu D, Op Den Buijs J, McEligot S, Dai Y, Entwistle RC, Salas C, Melton LJ, Bennet KE, Khosla S, Amin S (2011). Robust QCT/FEA models of proximal femur stiffness and fracture load during a sideways fall on the hip. Ann Biomed Eng.

[18] Schileo E, Balistreri L, Grassi L, Cristofolini L, Taddei F (2014). To what extent can linear finite element models of human femora predict failure under stance and fall loading configurations?. J Biomech.

[19] Dall’Ara E, Luisier B, Schmidt R, Kainberger F, Zysset P, Pahr D (2013). A nonlinear QCT-based finite element model validation study for the human femur tested in two configurations in vitro. Bone.

[20] Stromsoe K, Hoiseth A, Alho A, Kok WL (1995). Bending strength of the femur in relation to non-invasive bone mineral assessment. J Biomech.

[21] Cheng XG, Lowet G, Boonen S, Nicholson PH, Brys P, Nijs J, Dequeker J (1997). Assessment of the strength of proximal femur in vitro: relationship to femoral bone mineral density and femoral geometry. Bone.

[22] Eckstein F, Lochmuller EM, Lill CA, Kuhn V, Schneider E, Delling G, Muller R (2002). Bone strength at clinically relevant sites displays substantial heterogeneity and is best predicted from site-specific bone densitometry. J Bone Miner Res.

[23] Bousson V, Le Bras A, Roqueplan F (2006). Volumetric quantitative computed tomography of the proximal femur: relationships linking geometric and densitometric variables to bone strength. Role for compact bone. Osteoporos Int.

[24] Hazrati Marangalou J, Ito K, Cataldi M, Taddei F, van Rietbergen B (2013). A novel approach to estimate trabecular bone anisotropy using a database approach. J Biomech.

[25] Taghizadeh E, Reyes M, Zysset P, Latypova A, Terrier A, Buchler P (2016). Biomechanical role of bone anisotropy estimated on clinical CT scans by image registration. Ann Biomed Eng.

[26] Bousson VD, Adams J, Engelke K, Aout M, Cohen-Solal M, Bergot C, Haguenauer D, Goldberg D, Champion K, Aksouh R, Vicaut E, Laredo JD (2011). In vivo discrimination of hip fracture with quantitative computed tomography: results from the prospective European femur fracture study (EFFECT). J Bone Miner Res.

[27] Yang L, Udall WJ, McCloskey EV, Eastell R (2014). Distribution of bone density and cortical thickness in the proximal femur and their association with hip fracture in postmenopausal women: a quantitative computed tomography study. Osteoporos Int.

[28] Taddei F, Falcinelli C, Balistreri L, Henys P, Baruffaldi F, Sigurdsson S, Gudnason V, Harris TB, Dietzel R, Armbrecht G, Boutroy S, Schileo E (2016). Left-right differences in the proximal femur’s strength of post-menopausal women: a multicentric finite element study. Osteoporos Int.

[29] Viceconti M, Bellingeri L, Cristofolini L, Toni A (1998). A comparative study on different methods of automatic mesh generation of human femurs. Med Eng Phys.

[30] Bardyn T, Reyes M, Larrea X, Büchler P, Miller K, Nielsen PMF (2010). Influence of smoothing on voxel-based mesh accuracy in micro-finite element. Computational biomechanics for medicine.

[31] Zysset P, Pahr D, Engelke K, Genant HK, McClung MR, Kendler DL, Recknor C, Kinzl M, Schwiedrzik J, Museyko O, Wang A, Libanati C (2015). Comparison of proximal femur and vertebral body strength improvements in the FREEDOM trial using an alternative finite element methodology. Bone.

[32] Rossman T, Kushvaha V, Dragomir-Daescu D (2016). QCT/FEA predictions of femoral stiffness are strongly affected by boundary condition modeling. Comput Methods Biomech Biomed Engin.

[33] Qasim M, Altai Z, Li X, Viceconti M. Clinical pathway for osteoporotic hip fracture risk assessment using patient-specific finite element models. 23rd European Society of Biomechanics conference - Seville, Spain, 2017.

[34] Keaveny TM, Kopperdahl DL, Melton LJ, Hoffmann PF, Amin S, Riggs BL, Khosla S (2010). Age-dependence of femoral strength in white women and men. J Bone Miner Res.

[35] Agten CA, Ramme AJ, Kang S, Honig S, Chang G (2017). Cost-effectiveness of virtual bone strength testing in osteoporosis screening programs for postmenopausal women in the United States. Radiology.

[36] Walpole SC, Prieto-Merino D, Edwards P, Cleland J, Stevens G, Roberts I (2012). The weight of nations: an estimation of adult human biomass. BMC Public Health.

[37] Leal J, Gray AM, Prieto-Alhambra D, Arden NK, Cooper C, Javaid MK, Judge A, group REs (2016). Impact of hip fracture on hospital care costs: a population-based study. Osteoporos Int.

[38] National Institute for Health and Clinical Excellence (NICE) Guideline N. 124 (2011). The Management of Hip Fracture in Adults.

[39] Tosteson AN, Gabriel SE, Grove MR, Moncur MM, Kneeland TS, Melton LJ (2001). Impact of hip and vertebral fractures on quality-adjusted life years. Osteoporos Int.

[40] Anitha D, Subburaj K, Mei K, Kopp FK, Foehr P, Noel PB, Kirschke JS, Baum T (2016). Effects of dose reduction on bone strength prediction using finite element analysis. Sci Rep.

[41] Cummings SR, Black DM, Rubin SM (1989). Lifetime risks of hip, Colles’, or vertebral fracture and coronary heart disease among white postmenopausal women. Arch Intern Med.

[42] Crandall CJ, Newberry SJ, Diamant A, Lim YW, Gellad WF, Booth MJ, Motala A, Shekelle PG (2014). Comparative effectiveness of pharmacologic treatments to prevent fractures: an updated systematic review. Ann Intern Med.

